# The NtrYX Two-Component System Regulates the Bacterial Cell Envelope

**DOI:** 10.1128/mBio.00957-20

**Published:** 2020-05-19

**Authors:** Kimberly C. Lemmer, François Alberge, Kevin S. Myers, Alice C. Dohnalkova, Ryan E. Schaub, Jonathan D. Lenz, Saheed Imam, Joseph P. Dillard, Daniel R. Noguera, Timothy J. Donohue

**Affiliations:** a Wisconsin Energy Institute, Great Lakes Bioenergy Research Center, University of Wisconsin—Madison, Madison, Wisconsin, USA; b Pacific Northwest National Laboratory, Environmental Molecular Sciences Laboratory, Richland, Washington, USA; c Department of Medical Microbiology and Immunology, University of Wisconsin—Madison, Madison, Wisconsin, USA; d Department of Bacteriology, University of Wisconsin—Madison, Madison, Wisconsin, USA; e Department of Civil and Environmental Engineering, University of Wisconsin—Madison, Madison, Wisconsin, USA; University of Washington

**Keywords:** *Rhodobacter*, cell division, cell envelope, lipopolysaccharide, peptidoglycan, periplasm, proteobacteria, two-component system

## Abstract

Activity of the NtrYX two-component system has been associated with important processes in diverse bacteria, ranging from symbiosis to nitrogen and energy metabolism. In the facultative alphaproteobacterium Rhodobacter sphaeroides, loss of the two-component system NtrYX results in increased lipid production and sensitivity to some known cell envelope-active compounds. In this study, we show that NtrYX directly controls multiple properties of the cell envelope. We find that the response regulator NtrX binds upstream of cell envelope genes, including those involved in peptidoglycan biosynthesis and modification and in cell division. We show that loss of NtrYX impacts the cellular levels of peptidoglycan precursors and lipopolysaccharide and alters cell envelope structure, increasing cell length and the thickness of the periplasm. Cell envelope function is also disrupted in the absence of NtrYX, resulting in increased outer membrane permeability. Based on the properties of R. sphaeroides cells lacking NtrYX and the target genes under direct control of this two-component system, we propose that NtrYX plays a previously undescribed, and potentially conserved, role in the assembly, structure, and function of the cell envelope in a variety of bacteria.

## Abstract (precis)

The bacterial cell envelope provides many important functions. It protects cells from harsh environments, serves as a selective permeability barrier, houses bioenergetic functions, defines sensitivity to antibacterial agents, and plays a crucial role in biofilm formation, symbiosis, and virulence. Despite the important roles of this cellular compartment, we lack a detailed understanding of the biosynthesis and remodeling of the cell envelope. Here, we report that the R. sphaeroides two-component signaling system NtrYX is a previously undescribed regulator of cell envelope processes, providing evidence that it is directly involved in controlling transcription of genes involved in cell envelope assembly, structure, and function in this and possibly other bacteria. Thus, our data report on a newly discovered process used by bacteria to assemble and remodel the cell envelope.

## INTRODUCTION

The bacterial cell envelope is a multifunctional compartment that defines the structure of the cell, allows selective passage of nutrients in and waste products out, and protects it from external environments (1). The Gram‐negative cell envelope contains an outer membrane (OM), a peptidoglycan (PG) layer, a cytoplasmic or inner membrane (IM), and a periplasm (1). The composition and functions of the cell envelope are known to be modified in response to internal and environmental cues in many bacteria (2, 3).

Two-component systems (TCSs) are signal transduction pathways that enable bacteria to detect internal and environmental signals and link them with appropriate genetic and biochemical programs for adaptation and survival (4). Prototypical TCSs contain a histidine kinase (HK) that autophosphorylates upon perception of a stimulus and transfers the phosphoryl group to a cognate response regulator (RR), which is then able to perform output functions, most commonly the modulation of gene expression (4). There are many examples of TCSs that regulate cell envelope functions in response to a variety of signals (e.g., WalRK, MtrAB) (5).

The NtrYX TCS is found in many proteobacteria, including numerous members of the alpha- and betaproteobacteria (6), and has been implicated in a variety of cellular processes. In Brucella abortus, activity of the HK NtrY responds to low O_2_ tension, and loss of NtrYX reduces expression of bioenergetic enzymes important for survival in low O_2_ (7, 8). In other species, the NtrYX TCS has also been implicated in regulating low-O_2_ or anaerobic processes, including biofilm formation (6, 9), symbiotic root nodulation and nitrogen fixation (9–12), pathogen intracellular survival (7, 13), and photosynthesis (14). In addition, in Sinorhizobium meliloti, loss of NtrYX results in increased exopolysaccharide synthesis, decreased salt and detergent tolerance, and altered cell shape (9, 15). Direct regulation of gene expression by the NtrC family member NtrX has been shown only for the nitrate metabolism genes *narK* and *narX* in Herbaspirillum seropedicae (16), the amino acid metabolism genes *putA* and *glnA* in Ehrlichia chaffeensis (13), the photosynthesis *puf* promoter in Rhodobacter capsulatus (14), and the *ntrYX* operon in B. abortus (17). Thus, it has been difficult to associate the myriad phenotypes reported for NtrYX mutants with a direct role of NtrX in regulating individual genes.

In the Gram-negative bacterium Rhodobacter sphaeroides, adaptation to low O_2_ tension requires cell envelope remodeling (18). We previously showed that loss of the NtrYX TCS increased lipid content (19), a cell envelope change that occurs under low O_2_ conditions in R. sphaeroides (20). We also found that NtrYX mutants were sensitive to compounds that target the cell envelope (19). Together, these observations suggest a link between NtrYX activity and cell envelope properties.

Here, we show that R. sphaeroides NtrX is a direct transcriptional regulator of genes involved in cell envelope structure and function. Loss of NtrYX increases cellular lipopolysaccharide (LPS) content, alters cell morphology, increases OM permeability, changes the abundance of cytoplasmic PG precursors, and increases the thickness of the periplasm. We conclude that R. sphaeroides NtrYX has a direct role in regulating cell envelope properties.

## RESULTS

### Identifying direct targets for the NtrYX TCS.

**FIG 1:**
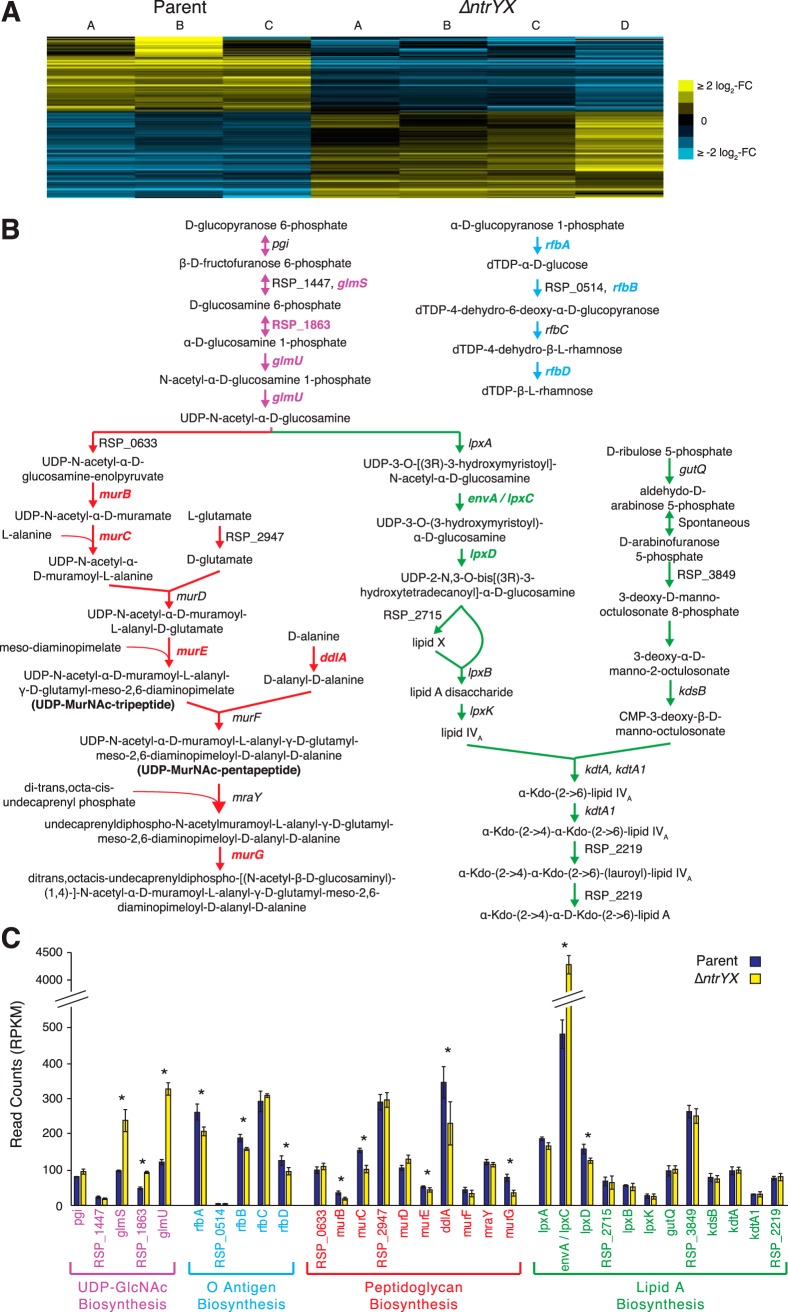
Comparison of transcript levels in parent versus Δ*ntrYX* strains. (A) Heatmap showing the transcripts found to be differentially expressed in Δ*ntrYX* cells compared to the parent strain under aerobic growth conditions (edgeR FDR ≤ 0.05, fold change ≥ 2.0). Biological replicates are labeled A to C for the parent strain and A to D for the Δ*ntrYX* strain. Each gene value has been mean centered across all experiments; yellow indicates an increased transcript level relative to the mean, while blue indicates a decreased transcript level relative to the mean. The scale bar to the right shows the log_2_ fold change (FC) scale for both colors. (B) Biochemical pathways involved in biosynthesis of UDP-*N*-acetylglucosamine (UDP-GlcNAc) (purple arrows), O antigen (blue arrows), peptidoglycan (red arrows), and lipid A (green arrows). Gene names in bold font and color are those with a significant change in expression in Δ*ntrYX* cells compared to the parent strain (edgeR FDR ≤ 0.05). Metabolites in bold font were measured in experiments described in Fig. 3 (UDP-MurNAc-tripeptide and -pentapeptide). (C) Transcript levels (RPKM normalized read counts) for genes involved in the four pathways from panel B in the parent strain (dark blue) and Δ*ntrYX* strain (yellow). Error bars indicate standard deviations. Genes with a significant difference in transcript levels between the strains (edgeR FDR ≤ 0.05) are indicated by an asterisk. Sections of panels B and C are shown in color (blue, red, orange, and green) to indicate genes that encode enzymes in the indicated biosynthetic pathways.

To investigate how loss of R. sphaeroides NtrYX may lead to the previously observed effects on cell envelope content and function (19), we used genomic analyses to identify potential pathways that were altered by loss of this TCS. Our previous studies were performed in strains deficient at synthesizing polyhydroxybutyrate (PHB), Δ0382 and ΔNtrYXΔ0382 (19); therefore, we continued to analyze these strains, which herein are referred to as parent and Δ*ntrYX*, respectively. We used genome-wide RNA sequencing (RNA-seq) to compare transcript abundance in parent and Δ*ntrYX* strains grown under aerobic conditions. We found 81 total genes with at least a twofold increase in transcript abundance in Δ*ntrYX* cells compared to the parent (false discovery rate [FDR] ≤ 0.05) and 70 total genes with at least a twofold decrease in Δ*ntrYX* cells compared to the parent (FDR ≤ 0.05) (Fig. 1A; see also Data Set S1 in the supplemental material).

10.1128/mBio.00957-20.8 DATA SET S1 RNA-seq data of parent and Δ*ntrYX* strains. Download Data Set S1, XLSX file, 0.7 MB. Copyright © 2020 Lemmer et al. 2020 Lemmer et al. This content is distributed under the terms of the Creative Commons Attribution 4.0 International license.

To gain insight into the cellular pathways altered by loss of NtrYX, we analyzed transcripts with a significant change in abundance in the Δ*ntrYX* strain compared to the parent strain for enrichment of any pathway or Gene Ontology term. We found that genes encoding PG and UDP-*N*-acetylglucosamine (UDP-GlcNAc), a precursor of PG and LPS, biosynthesis were enriched among genes with significant changes in transcript abundance in the Δ*ntrYX* strain compared to the parent strain (Fig. 1B and C). We also found significant changes in transcript abundance for genes encoding steps in the synthesis of LPS components O antigen and lipid A, including a large increase in mRNA derived from *envA/lpxC*, encoding the first committed step in lipid A biosynthesis, in the Δ*ntrYX* strain compared to the parent strain (Fig. 1B and C). Combined, these changes in transcript abundance suggest that there is a connection between the R. sphaeroides TCS NtrYX and biosynthesis of PG and LPS.

To ask whether genes encoding any of these cell envelope-related pathways are directly regulated by R. sphaeroides NtrYX, we used genome-scale chromatin immunoprecipitation sequencing (ChIP-seq) to identify binding sites of the transcription factor NtrX; this analysis identified six high-confidence NtrX binding sites in the genome (Table 1). One R. sphaeroides NtrX binding site is upstream of its own operon, suggesting that it autoregulates *ntrYX* transcription. The other five NtrX binding sites are upstream of genes and operons encoding functions related to the cell envelope, including PG and extracellular polysaccharide synthesis, cell wall remodeling enzymes, a cytoskeletal protein, a putative lipoprotein, and cell division proteins (Table 1). This predicts that NtrX plays a direct role in regulating transcription of some of the cell envelope pathways identified when comparing transcript levels between Δ*ntrYX* and parent cells. Indeed, the transcriptomic analysis of the parent and Δ*ntrYX* cells showed significant changes in RNA levels (FDR ≤ 0.05) in genes downstream of many identified NtrX binding sites (Table 1). Also, two of the identified NtrX target operons (*mraZ-mraY* and *ddlA-ftsA*) are part of the highly conserved division cell wall (*dcw*) gene cluster, containing genes for PG synthesis and cell division. Indeed, of the 17 conserved genes of the *dcw* cluster that are present in R. sphaeroides, transcripts for 12 of them were significantly less abundant (FDR ≤ 0.05) in the Δ*ntrYX* strain, while transcripts for the last gene in the cluster, *envA/lpxC*, were elevated in cells lacking NtrYX (Data Set S1).

**TABLE 1:** NtrX binding sites identified by ChIP-seq

Peak start^*a*^ (bp)	Peak stop^*a*^ (bp)	ChIP fold enrichment (IP/input)	Downstream gene			Transcript log_2_ fold change (Δ*ntrYX*/parent)	Transcript change FDR
			ID^*b*^	Name	Product annotation		
458,400	458,999	8.5 ± 1.5	RSP_1860	RSP_1860	Cell wall hydrolase, CwlJ-like	1.12	4.2 × 10^−5^

1,472,000	1,472,399	4.2 ± 0.5	RSP_2839	*ntrY*	Two-component system sensor histidine kinase NtrY	ND^*c*^	2.4 × 10^−7^
			RSP_2840	*ntrX*	Two-component system response regulator NtrX	ND^*c*^	2.4 × 10^−7^

2,064,600^*d*^	2,065,100^*d*^	2.5 ± 0.0^*d*^	RSP_0335	RSP_0335	Diguanylate cyclase/phosphodiesterase	−0.28	1.7 × 10^−2^

2,064,600^*d*^	2,065,100^*d*^	2.5 ± 0.0^*d*^	RSP_0334	RSP_0334	Hypothetical protein with lipoprotein lipid attachment site	−0.80	6.2 × 10^−4^
			RSP_0333	RSP_0333	Cellulose synthase catalytic unit (UDP-forming)	−0.68	1.9 × 10^−4^
			RSP_0332	RSP_0332	Cellulose synthase subunit	−0.42	2.7 × 10^−3^

1,661,000	1,661,399	2.6 ± 0.2	RSP_2974	RSP_2974	Murein dd-endopeptidase MepM/murein hydrolase activator NlpD	0.34	0.11
			RSP_2975	RSP_2975	Cytoskeletal protein CcmA (bactofilin family)	0.06	0.99

694,110	695,200	2.1 ± 0.1	RSP_2095	*mraZ*	MraZ protein	−0.63	2.4 × 10^−3^
			RSP_6038	*rsmH/mraW*	16S rRNA (cytosine1402-N4)-methyltransferase	−0.61	2.2 × 10^−3^
			RSP_2097	RSP_2097	Hypothetical protein (putative FtsL)	−0.52	1.8 × 10^−2^
			RSP_2098	*ftsI*	Cell division protein FtsI (penicillin-binding protein 3)	−0.45	3.0 × 10^−3^
			RSP_2099	*murE*	UDP-*N*-acetylmuramoylalanyl-d-glutamate− 2,6-diaminopimelate ligase	−0.27	3.5 × 10^-−2^
			RSP_2100	*murF*	UDP-*N*-acetylmuramoyl-tripeptide−d-alanyl-d-alanine ligase	−0.38	8.7 × 10^−2^
			RSP_2101	*mraY*	Phospho-*N*-acetylmuramoyl-pentapeptide-transferase	−0.09	0.26

711,380	712,030	2.8 ± 1.1	RSP_2111	*ddlA*	d-Alanine-d-alanine ligase	−0.58	2.7 × 10^−2^
			RSP_2112	*ftsQ*	Cell division protein FtsQ	−1.07	1.4 × 10^−3^
			RSP_2113	*ftsA*	Cell division protein FtsA	−1.04	2.7 × 10^−5^

a All peak locations are on chromosome 1.

b ID, identifier.

c ND, not defined, because in the Δ*ntrYX* strain, the transcript level was zero.

d This ChIP-seq peak was located in the shared upstream intergenomic region between RSP_0335 and RSP_0334.

### Loss of NtrYX leads to altered cell morphology and PG precursor abundance.

**FIG 2:**
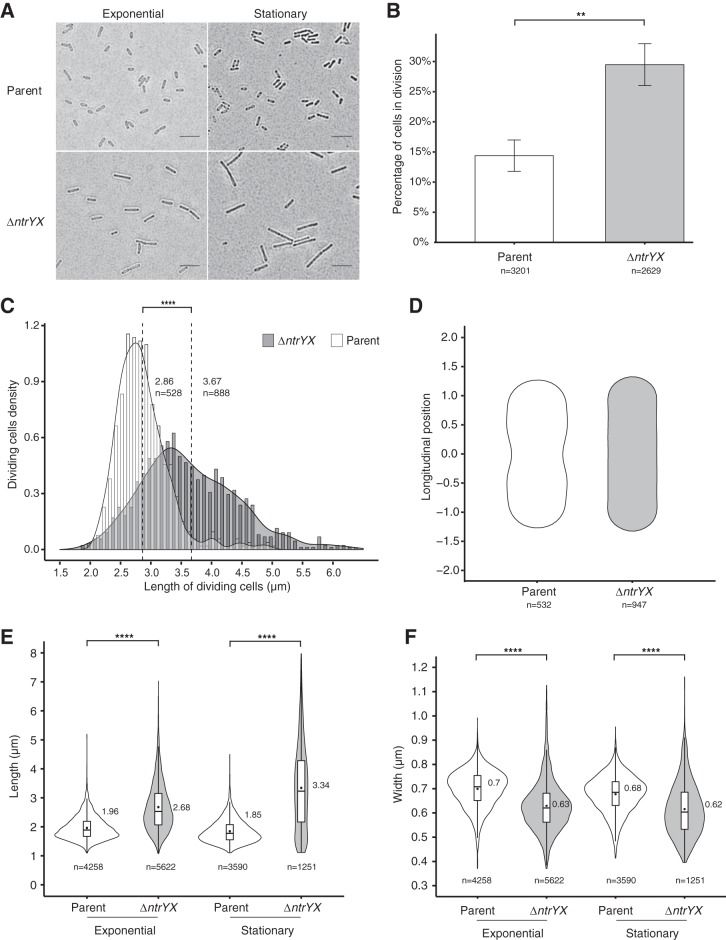
Analysis of cell morphology in aerobically grown parent and Δ*ntrYX* cells. (A) Bright-field microscopy images of cells in exponential and stationary growth phases. Bars, 5 μm. (B) Percentage of cells scored as dividing during exponential growth phase. Cells were scored as dividing if they contained a detectable mid-cell constriction in the membrane using the fluorescent membrane dye FM-64 and, if that was ambiguous, one or two DNA foci at this mid-cell position. Examples of a stained dividing and nondividing cell are shown in Fig. S1 in the supplemental material. (C) Histogram and probability density plot representing the length distribution of cells scored as dividing in parent (white bars) and Δ*ntrYX* (dark gray bars) cultures. The dashed lines represent the respective means, with values and numbers of cells (*n*) used for this analysis listed to the right of each line. (D) Representation of the mean shape of parent (white) and Δ*ntrYX* (gray) cells with a length ranging from 2.3 to 3 μm for each strain. Coordinates were obtained with MicrobeJ using the indicated number (*n*) of bacteria. (E and F) Violin plots of the length (E) and width (F) of parent (white) or Δ*ntrYX* (gray) cells during exponential or stationary growth phase. Cells with a fully formed division septum were excluded. The outer boundary of the plots represents the probability density for all the data in the sample. Box plots are shown inside the violin plots, with lower and upper boundaries of the box representing the first and third quartile, respectively. The horizontal bar inside the box represents the median. The black dots inside the box represent the mean, with the corresponding values to the side. The numbers of cells (*n*) analyzed from at least three different experiments are indicated below the plots. Unpaired *t* tests were used to compare the populations in panels B, C, E, and F with the asterisks indicating a significant *P* value as follows: **, *P* < 0.01; ****, *P* < 0.0001.

Since the above analysis indicated direct control by NtrYX on the *dcw* cluster, we used bright-field microscopy to look for effects of this TCS on cell morphology. We observed an increase in the occurrence of two cells end to end in Δ*ntrYX* cultures compared to parent cultures (Fig. 2A), suggesting that more of the exponential-phase Δ*ntrYX* cells had initiated cell division, but not yet separated. However, we did not observe the long chains of cells that are often associated with complete blocks in cell division (21). To investigate this further, we stained membranes and DNA (with FM4-64 and 4′,6′-diamidino-2-phenylindole [DAPI], respectively) to test for diagnostic features of cell division. Cells were scored as dividing if they contained a detectable mid-cell constriction in the membrane (the site of septum formation) and one or two DNA foci at this mid-cell position (see Fig. S1 in the supplemental material). Once there was a detectable pixel gap between the membranes of adjacent cells, they were scored as no longer dividing. This analysis scored ∼14% of the cells as dividing in parent cultures, compared with ∼29% in the Δ*ntrYX* cultures (Fig. 2B). To ask whether this apparent increase in dividing cells could be due to differences in the growth rate between the Δ*ntrYX* and parent strain, we measured doubling times and found that the Δ*ntrYX* cultures had a lower growth rate than the parent strains (2.9-h doubling time for the parent versus 4.0 h for the Δ*ntrYX* strain; Table S1). Thus, the observed increase in the percentage of Δ*ntrYX* cells that have initiated mid-cell septum formation does not reflect a higher growth rate of the mutant strain. Rather, this difference could reflect the fact that cells lacking NtrYX take longer to complete the process of cell division.

10.1128/mBio.00957-20.1 FIG S1 Representative images of a dividing (A to C) and a nondividing (D to F) Δ*ntrYX* cell. (A and D) Bright-field microscopy images in which segmentation and membrane constriction are detected by MicrobeJ. (B and E) Fluorescent membrane staining by FM4-64, which is used to confirm mid-cell constriction and the presence of the division septum. (C and F) Fluorescent DNA staining with DAPI, which is used to confirm the condensed DNA in dividing cells. The scale bars (A and D) correspond to 2 μm. Download FIG S1, EPS file, 0.5 MB. Copyright © 2020 Lemmer et al. 2020 Lemmer et al. This content is distributed under the terms of the Creative Commons Attribution 4.0 International license.

10.1128/mBio.00957-20.5 TABLE S1 Growth rates of parent and Δ*ntrYX* cultures. Download Table S1, PDF file, 0.02 MB. Copyright © 2020 Lemmer et al. 2020 Lemmer et al. This content is distributed under the terms of the Creative Commons Attribution 4.0 International license.

During this analysis, we also found that there was a difference in the mean cell length, pole to pole, of cells that contained detectable division septa between the Δ*ntrYX* and parent strains, with a mean length of 2.9 μm for the parent and 3.7 μm for cells lacking NtrYX (Fig. 2C). In the histograms of these measurements, the NtrYX mutant also has a wider distribution of length values, which could be due to either a delay in initiation or progression of division or both. To ask whether cells lacking NtrYX initiated septum formation at a longer length, we used MicrobeJ software (22) to plot the average cell shape for all cells 2.3 to 3.0 μm long for both strains. In the parent cells, a mid-cell constriction is seen at this cell length interval, showing that most of these cells have entered division phase (Fig. 2D). In contrast, a division septum is not observed in the Δ*ntrYX* cells in this same length interval (Fig. 2D). We also found that the mean cell length of exponential-phase cells increases from 2.0 μm in the parent cells to 2.7 μm in the Δ*ntrYX* cells and from 1.9 μm to 3.3 μm, respectively, in stationary phase, while the mean cell width decreased from 0.70 μm to 0.63 μm in growth phase and from 0.68 μm to 0.62 μm in stationary phase (Fig. 2E and F). These changes in cell morphology also result in a significant increase in longitudinal-section area in Δ*ntrYX* cells compared to parent cells (Fig. S2). Thus, on average, the Δ*ntrYX* cells are longer, narrower, and larger than the parent cells and initiate septum formation at a longer average length.

10.1128/mBio.00957-20.2 FIG S2 Violin plots of cell length, width, and area measurements for aerobically grown parent, HLM01, HLM02, and Δ*ntrYX* cells in exponential and stationary growth phases. HLM01 and HLM02 strains have transposon insertions disrupting activity of the NtrYX TCS (K. C. Lemmer, W. Zhang, S. J. Langer, A. C. Dohnalkova, et al., mBio 8:e00513-17, 2017, https://doi.org/10.1128/mBio.00513-17). The outer boundary of the plots represents the probability density for all the data in the sample. Box plots are shown inside the violin plots, with lower and upper boundaries of the box representing the first and third quartile, respectively. The horizontal bar inside box represents the median. The black dots inside the box represent the mean, with the corresponding values to the side. Unpaired *t* tests were used to compare the value for each mutant to that of the parent, with statistical significance indicated as follows: ****, *P* < 0.0001; ns, not significant. Download FIG S2, PDF file, 1.0 MB. Copyright © 2020 Lemmer et al. 2020 Lemmer et al. This content is distributed under the terms of the Creative Commons Attribution 4.0 International license.

Since cell shape is largely determined by the PG layer (23, 24), we asked whether Δ*ntrYX* cells had changes in the composition of this cell wall polymer. To do this, we performed muropeptide analysis of sacculi isolated from exponential and stationary-phase parent and Δ*ntrYX* cultures. We observed muropeptide differences between exponential and stationary phases in the parent strain (Fig. S3), as expected from analysis of other Gram-negative bacteria (25). However, we found no reproducible differences in the composition of the PG between the parent and Δ*ntrYX* strains in either growth phase (Fig. S3).

10.1128/mBio.00957-20.3 FIG S3 Chromatograms of PG fragments from parent and Δ*ntrYX* cells in exponential (A) and stationary (B) growth phase. Aerobic cultures (500 ml) were harvested in exponential (OD_600_ of ∼0.5) or stationary (OD_600_ >1.5) phase. Peptidoglycan sacculi were purified, digested with mutanolysin, and separated by high-performance liquid chromatography (HLPC) as described previously (R. E. Schaub and J. P. Dillard, Bio Protoc 7:e2438, 2017, https://doi.org/10.21769/BioProtoc.2438). Download FIG S3, EPS file, 0.8 MB. Copyright © 2020 Lemmer et al. 2020 Lemmer et al. This content is distributed under the terms of the Creative Commons Attribution 4.0 International license.

**FIG 3:**
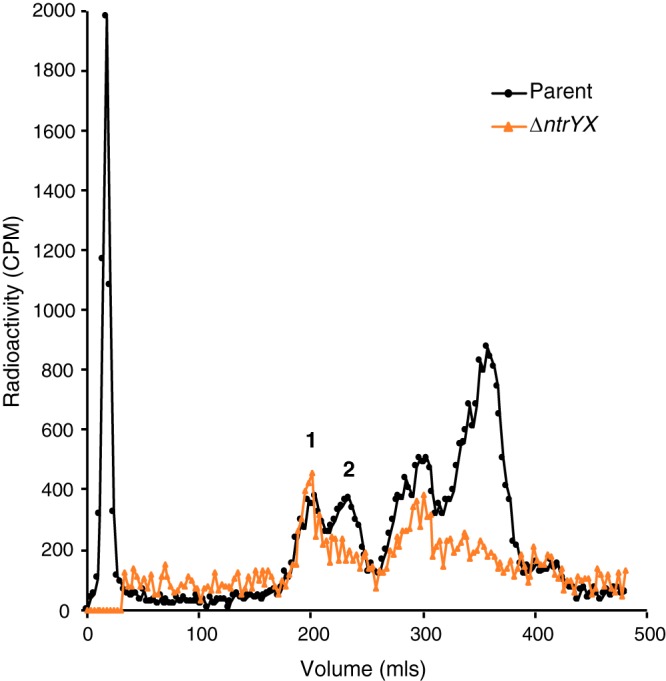
Analysis of soluble PG precursor abundance. Parent and Δ*ntrYX* aerobic cultures were pulse-labeled with [^3^H]glucosamine, and water-soluble PG precursors were collected during a chase period. Radiolabeled compounds were separated by size exclusion chromatography, and their radioactivity was measured by liquid scintillation counting. The identities of the indicated peaks (peaks 1 and 2) were confirmed by comparing retention time with those of known metabolites. Peak 1 is UDP-MurNAc-pentapeptide, and peak 2 is UDP-MurNAc-tripeptide. Precursors to the right of the labeled peaks are smaller in size than UDP-MurNAc-tripeptide, but we were not able to determine their identity.

The genome-wide transcript analysis revealed reduced RNA abundance for several genes encoding enzymes for cytoplasmic steps of PG precursor biosynthesis (*murB*, *murC*, *murE*, *murG*, and *ddlA*) (26) in the Δ*ntrYX* strain compared to the parent strain (Fig. 1B and C and Data Set S1). In addition, NtrX was found to bind upstream of two operons that encode PG precursor synthesis enzymes (MurE, MurF, MraY, and DdlA; Table 1). To ask whether loss of NtrYX led to altered levels of PG precursors, parent and Δ*ntrYX* cells were labeled with [^3^H]glucosamine, and after a chase with unlabeled glucosamine, the total (cytoplasmic and periplasmic) water-soluble PG was extracted by boiling, separated from the sacculus by centrifugation, and analyzed by size exclusion chromatography. Parent cells contained approximately equal amounts of UDP-MurNAc-tripeptide and UDP-MurNAc-pentapeptide (Fig. 3), the cytoplasmic products of MurE and MurF activity, respectively (26) (Fig. 1B). In contrast, Δ*ntrYX* cells contained significantly more UDP-MurNAc-pentapeptide than UDP-MurNAc-tripeptide (Fig. 3). This analysis identified higher levels of additional smaller PG fragments in both strains than previously reported in other species (27, 28). We also found that Δ*ntrYX* cells routinely incorporated less [^3^H]glucosamine than the parent cells, so we were not able to compare the absolute levels of these water-soluble PG precursors between strains. However, differences in the relative ratios of UDP-MurNAc-tripeptide and -pentapeptide demonstrate that PG metabolism is altered and that Δ*ntrYX* cells have decreased availability of the penultimate PG precursor compared to parent cells.

### Loss of NtrYX changes OM composition and function.

**FIG 4:**
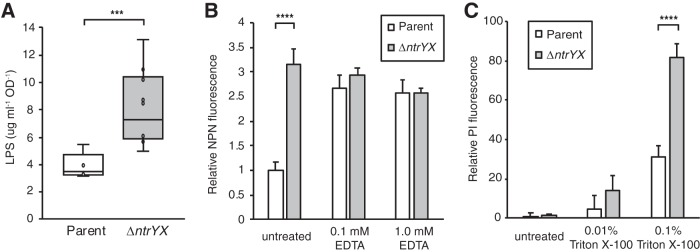
Analysis of OM composition and function. (A) Box plot of LPS levels in parent (white) and Δ*ntrYX* (gray) cells grown aerobically. The lower and upper boundaries of the boxes represent the first and third quartile, respectively. The horizontal bar inside box plots represents the median. The top and bottom whiskers extend to the maximum and minimum values, respectively. (B) *N*-Phenyl-1-naphthylamine (NPN) fluorescence as a reporter of outer membrane permeability. Parent (white) and Δ*ntrYX* (gray) aerobic cultures were incubated with NPN and, where indicated, EDTA as a known outer membrane permeabilizer. (C) Propidium iodide (PI) fluorescence as a reporter of membrane integrity and cell viability in parent (white) and Δ*ntrYX* (gray) aerobic cultures. For panels B and C, fluorescence intensity was measured on a 96-well plate reader. Error bars on these bar graphs indicate standard deviations. Data shown in all panels are from four or more independent cultures. Unpaired *t* tests were used to compare the populations with the asterisks indicating a significant *P* value as follows: ***, *P* < 0.001; ****, *P* < 0.0001.

The transcript and NtrX binding data predicted that there may be additional cell envelope changes between the parent and Δ*ntrYX* cells. For example, transcripts of genes for UDP-GlcNAc biosynthesis, a precursor for both PG and lipid A biosynthesis, were more abundant in the Δ*ntrYX* strain than in the parent strain (Fig. 1B and C). In addition, *envA/lpxC*, which encodes the first committed step of lipid A synthesis, was one of the most highly differentially expressed genes between the parent and Δ*ntrYX* cultures, with an 8.8-fold increase in transcript abundance in cells lacking NtrYX compared to parent cells (Fig. 1B and C). To ask whether LPS biosynthesis was affected by the loss of NtrYX, we measured total LPS in parent and Δ*ntrYX* cells. We found that the Δ*ntrYX* cells contained approximately twice as much LPS as the parent cells when normalized to culture optical density (OD) (Fig. 4A). For a control, we measured cellular protein, DNA, and RNA levels, and found no significant changes in the content of these macromolecules when normalized to culture OD (Table S2). As expected from previous studies (19), Δ*ntrYX* cells contained twice as much cellular phospholipid as parent cells (Table S2).

10.1128/mBio.00957-20.6 TABLE S2 Cell biomass composition (in micrograms/milliliter) of aerobically grown parent and *ΔntrYX* cultures, both normalized to an absorbance of 1 at 600 nm. Download Table S2, PDF file, 0.02 MB. Copyright © 2020 Lemmer et al. 2020 Lemmer et al. This content is distributed under the terms of the Creative Commons Attribution 4.0 International license.

Since LPS is known to contribute to the permeability barrier function of the OM of Gram-negative cells (29), we asked whether this aspect of cell envelope function was altered in Δ*ntrYX* cells. To do this, we used *N*-phenyl-1-naphthylamine (NPN), a nonpolar indicator that fluoresces in phospholipid environments, as a reporter of OM permeability. NPN is normally excluded from Gram-negative bacteria by the OM; however, when the integrity of this membrane is compromised, such as after treatment with the well-known permeabilizer EDTA, there is increased NPN fluorescence due to its enhanced transport across the OM (30). We observed increased NPN fluorescence in *ΔntrYX* cells compared to parent cells (Fig. 4B), suggesting that OM permeability is increased in the mutant. We found that EDTA increases NPN fluorescence in the parent cells; however, there is no increase in NPN fluorescence observed when *ΔntrYX* cells are treated with the same concentration of EDTA (Fig. 4B). Thus, although *ΔntrYX* cells have an increase in LPS content, the OM seems less functional as a permeability barrier. These observations are consistent with our previous finding that NtrYX mutants are more sensitive to growth inhibition by EDTA treatment than parent cultures, as well as by drugs (rifampin, rifaximin, erythromycin, and clarithromycin) that are normally excluded by the wild-type OM (19). To test that the increase in NPN florescence was not due to an increase in dead or lysing cells, we asked whether there was a difference in staining by the membrane-impermeant nucleic acid stain propidium iodide (PI) between the parent and *ΔntrYX* cultures. We did not observe a significant increase in PI staining in the *ΔntrYX* cultures compared to the parent cultures (Fig. 4C), supporting the conclusions that the inner membranes of *ΔntrYX* cells remain largely intact in NtrYX cells and that the increased permeability in this strain is localized to the OM.

### Loss of NtrYX increases periplasm thickness.

**FIG 5:**
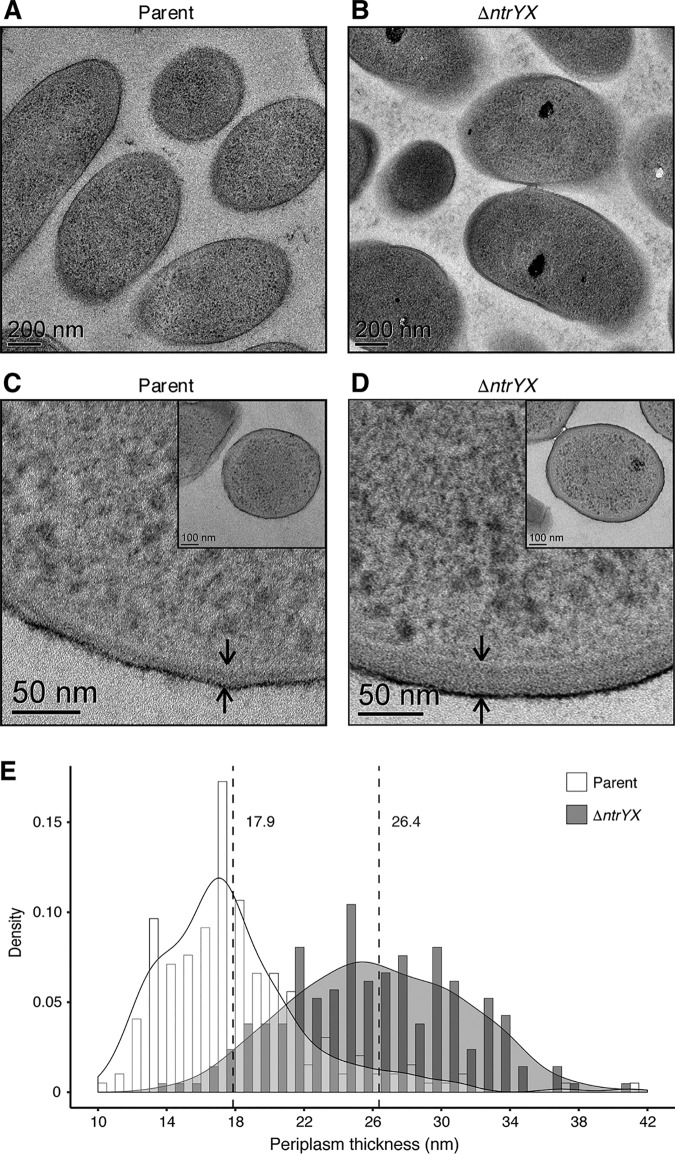
Analysis of the ultrastructure of the cell envelope. (A and B) TEM micrographs of thin sections of parent and Δ*ntrYX* cells grown aerobically. (C and D) Representative TEM cross sections used to measure the distance between the IM and OM. Arrows indicate the OM (black ring) and IM (white ring); the distance between these structures was used to determine the periplasm thickness. Insets show the whole-cell view for each representative image. (E) Histogram and probability density plot of the periplasm thickness measurements from analyzing TEM cross sections of parent (white bars) and Δ*ntrYX* (dark gray bars) cells. Dashed lines represent the respective means with values listed to the right of each line. An unpaired *t* test was used to compare the means with a *P* value of <0.0001.

To test for other changes in cell envelope morphology in cells lacking NtrYX, we used transmission electron microscopy (TEM) to probe the structure of parent and *ΔntrYX* cells. TEM thin sections showed similar intracellular ultrastructure in the parent and *ΔntrYX* cells, but cells lacking NtrYX appeared to have thickened cell envelopes (Fig. 5A and B). Indeed, using high-resolution imaging of transverse sections to measure the distance between the IM and OM (Fig. 5C and D), the *ΔntrYX* cells had a 47% increase in the mean periplasm thickness, from 17.9 nm in parent cells to 26.4 nm in cells lacking NtrYX (Fig. 5C to E).

### Other possible roles for R. sphaeroides NtrYX.

We also asked whether the R. sphaeroides *ΔntrYX* mutant had phenotypes associated with loss of NtrYX in other species (nitrogen assimilation or fixation, expression of low-oxygen or anaerobically induced genes [7, 10, 14, 16, 31]). To test whether loss of NtrYX affects N_2_ fixation, we grew parent and *ΔntrYX* strains under anaerobic photoheterotrophic conditions with ammonia or N_2_ as the nitrogen source. The *ΔntrYX* mutant grew under both conditions, albeit with longer doubling times than those for the parent, similar to the slower growth observed under aerobic conditions (Table S1), suggesting that loss of NtrYX does not cause a specific impairment in N_2_-fixing ability. In addition, the *ΔntrYX* mutant was able to grow aerobically and anaerobically with all nitrogen sources tested (ammonia, urea, glutamate) (Table S3). Finally, among the 151 differentially expressed transcripts that we identified comparing the parent and *ΔntrYX* strains, there were none encoding nitrogen metabolism or fixation functions (Data Set S1).

10.1128/mBio.00957-20.7 TABLE S3 Growth of parent and Δ*ntrYX* strains with different nitrogen sources. Download Table S3, PDF file, 0.03 MB. Copyright © 2020 Lemmer et al. 2020 Lemmer et al. This content is distributed under the terms of the Creative Commons Attribution 4.0 International license.

In another purple nonsulfur bacterium, R. capsulatus, NtrX has been reported to directly repress transcription of photosynthesis genes (14). Indeed, the R. sphaeroides *ΔntrYX* mutant had ∼2- to 3-fold-increased levels of transcripts for many photosynthesis genes compared to the parent when grown under aerobic conditions (where expression of these genes is normally low) (Data Set S1). However, we did not detect NtrX binding at any promoters for any known photosynthesis genes (Table 1 and Data Set S2), and although the transcript abundance for these genes in aerobically grown *ΔntrYX* cells is elevated, these RNA levels are much lower than those observed under low-O_2_ or anaerobic conditions. In addition, absorbance scans of *ΔntrYX* and parent cultures found no significant light-absorbing peaks that are diagnostic of R. sphaeroides pigment-protein complexes of the photosynthetic membrane (Fig. S4). Thus, our data predict that R. sphaeroides NtrX is not a direct repressor of photosynthetic gene expression under the conditions we tested.

10.1128/mBio.00957-20.4 FIG S4 Whole-cell absorbance scans of parent and Δ*ntrYX* aerobic cultures. The absence of peaks for all of the cultures show the lack of detectable photosynthetic pigment-protein complexes. Spectra were scaled to an absorbance of 1 at 680 nm to normalize for cell density and then staggered vertically for presentation of multiple curves on one axis. Two representative scans are shown for each strain. Download FIG S4, PDF file, 0.3 MB. Copyright © 2020 Lemmer et al. 2020 Lemmer et al. This content is distributed under the terms of the Creative Commons Attribution 4.0 International license.

10.1128/mBio.00957-20.9 DATA SET S2 ChIP-seq peaks identified for NtrX in replicate experiments. Download Data Set S2, XLSX file, 0.01 MB. Copyright © 2020 Lemmer et al. 2020 Lemmer et al. This content is distributed under the terms of the Creative Commons Attribution 4.0 International license.

## DISCUSSION

The bacterial cell envelope provides many important functions. It interacts with and protects cells from external environments, contributes to cell morphology, serves as a selective permeability barrier, houses bioenergetic and signaling functions, defines the sensitivity to antibacterial agents, and plays a crucial role in biofilm formation, symbiosis, and virulence (1). Despite the important roles of this cellular compartment, we lack a detailed understanding of the biosynthesis and remodeling of the cell envelope.

In this study, we show that the R. sphaeroides NtrYX TCS is directly involved in bacterial cell envelope assembly, structure, and function. We used genome-wide transcript and NtrX binding data to define the direct transcriptional targets of this RR, uncovering a role for NtrYX as a direct regulator of several aspects of cell envelope biology. We performed the current study in a strain deficient in synthesizing PHB; however, we have not observed any phenotypic differences in NtrYX mutant strains with and without an intact PHB synthesis pathway. Below, we summarize how our genome-scale data sets led to biochemical and imaging studies to demonstrate a set of previously unknown OM, periplasm, and other cell envelope changes associated with the loss of the NtrYX TCS.

### Direct targets of NtrX.

Using ChIP-seq, we found that R. sphaeroides NtrX bound upstream of the *ntrYX* operon, suggesting that it is autoregulatory similar to the situation in B. abortus (17). However, genes downstream of the other NtrX binding sites predict previously unreported targets for this TCS (Table 1). Transcript abundance of several NtrX target operons was lower in the NtrYX mutant than in parent cells (RSP_0335, RSP_0334-RSP_0332, *mraZ-mraY*, and *ddlA-ftsA*), suggesting that, like other NtrC family members (32), R. sphaeroides NtrX acts as an activator. In addition, transcript abundance of one direct NtrX target (RSP_1860) was increased in cells lacking NtrYX, so it is possible that NtrX also acts as a repressor of transcription, as was suggested for the *ntrY* promoter of B. abortus (17).

### Regulation of cell wall functions by NtrYX.

Many of the genes that we identified as direct targets of NtrX (Table 1) encode homologues of enzymes that act in PG synthesis (*murE*, *murF*, *mraY*, *ddlA*, *ftsI*) or hydrolysis (RSP_1860, RSP_2974) in other bacteria. These direct targets of NtrX lead to the hypothesis that cells lacking NtrYX would exhibit phenotypes that are associated with alterations in PG synthesis and/or cleavage (23). Consistent with this hypothesis, we observed a change in cell shape (longer and narrower) in cells lacking NtrYX compared to parent cells. While previous analysis of other R. sphaeroides NtrYX mutants by different assays did not show significant differences in cell length (19), analysis of strains containing either transposon insertions or in-frame deletions of *ntrYX* in this study revealed significant changes in cell shape between parent and NtrYX mutant cells (see Fig. S2 in the supplemental material). The specific functions of the putative PG hydrolases RSP_1860 and RSP_2974 have not been characterized in this bacterium, but cell wall hydrolases are known to affect cell growth and maintenance of cell shape in other species (2, 33), so it is possible that the observed changes in cell shape in NtrYX mutants are due to changes in the PG sacculus that we were not able to detect by muropeptide analysis (Fig. S3). However, homologues of other direct targets of NtrX have also been shown to affect cell shape in other bacteria, including RSP_2975 (the predicted cytoskeletal protein CcmA) (34, 35), and the cell division genes *ftsA*, *ftsQ*, *ftsL*, and *ftsI*.

In addition, we found that cells lacking NtrYX have decreased levels of the predicted penultimate cytoplasmic PG precursor UDP-MurNAc-tripeptide compared to parent cells. One explanation for the change in this PG precursor is the decreased levels of RNA encoding enzymes for PG precursor synthesis (*murB*, *murC*, *murE*, *murG*, *ddlA*) in cells lacking NtrYX. If these lower transcript levels limit the abundance of PG biosynthetic enzymes, it could decrease flux through this part of the pathway. Concurrently, maintained demand for the final PG precursor UDP-MurNAc-pentapeptide could cause rapid turnover of UDP-MurNAc-tripeptide to UDP-MurNAc-pentapeptide, as the transcript levels encoding MurF, which catalyzes this step, are not reduced (Fig. 1C). In addition, the predicted increased flux of UDP-GlcNAc into LPS synthesis (see below) could impose a further limitation on PG synthesis. Thus, we predict that some of the differences in cell size, shape, and growth rate in cells lacking NtrYX can be attributed to the impact of changes in PG precursors on synthesis of this important cell envelope polymer.

### Impact of NtrYX on cell division.

Bacterial cell division requires the coordinated synthesis and constriction of cell envelope components at the septum, and studies with other species have shown that proteins encoded by the conserved *dcw* gene cluster are important for these processes (36, 37). We show here that NtrX is a direct transcription activator of two operons within the *dcw* cluster (*mraZ-mraY* and *ddlA-ftsA*) (Table 1). These two NtrX-dependent operons are predicted to encode four gene products known to be essential for cell division in other organisms (FtsL, FtsI, FtsQ, and FtsA) (38), a transcription factor that may further regulate the *dcw* gene cluster (MraZ) (39), and enzymes for PG precursor synthesis described above.

Our analysis revealed an increased number of Δ*ntrYX* cells in a division state and septum formation at a longer and more varied cell length. These observations suggest that there could be a delay in initiation of cell division, as well as slowed progression of division, and are consistent with the decreased growth rate that we measured for Δ*ntrYX* cells. This is not surprising given the decreased transcript levels of four direct target genes of NtrX that encode proteins that localize to the division septum and are essential for cell division (FtsA, FtsQ, FtsI, and FtsL) (21, 38). In other organisms, loss of any of these *fts* genes blocks division completely, leading to long filamentous cells or loss of viability (21). However, decreased levels of these genes cause more moderate phenotypes, in particular, depletion of either *ftsA* or *ftsQ* leads to increased cell length in other bacteria (40, 41), similar to the longer length observed in Δ*ntrYX* cells that have twofold lower levels of *ftsA* and *ftsQ* transcripts (Table 1). Thus, it is likely that direct regulation of cell division genes in the *dcw* cluster contributes to the slowed initiation and progression of cell division, decreased grown rate, and altered cell shape of the NtrYX mutant. In addition, the effects of the loss of NtrYX on PG synthesis and hydrolysis, discussed above, could contribute to the observed effects on cell division, as these processes are necessary during septum formation and cell separation (38).

### Evidence for regulation of the cell envelope by NtrYX in other bacteria.

**FIG 6:**
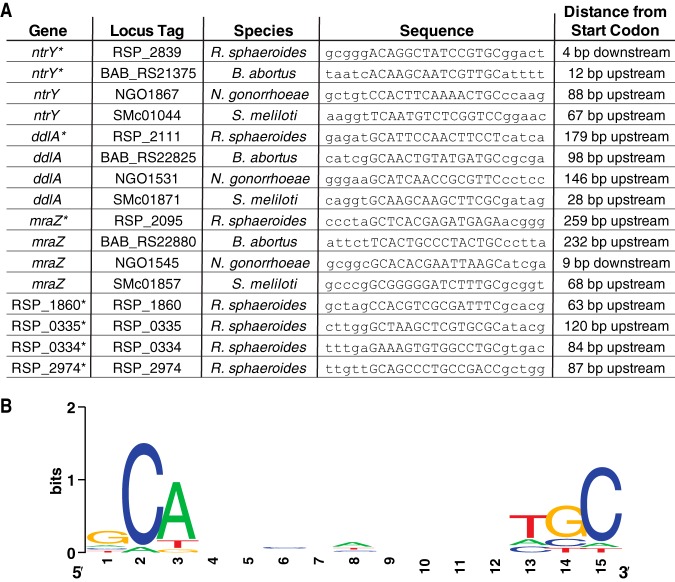
Prediction of NtrX binding sites upstream of predicted cell envelope operons in R. sphaeroides, B. abortus, N. gonorrhoeae, and S. meliloti. (A) Summary of the predicted NtrX binding sites. Regions known to bind NtrX are indicated with an asterisk. Homologs of *ntrY*, *ddlA*, and *mraZ* are included for B. abortus, N. gonorrhoeae, and S. meliloti, as indicated. The predicted NtrX binding site is in capital letters within each sequence, and the distance listed is from the end of the predicted NtrX binding site to the start codon of the indicated gene. (B) Sequence logo of the NtrX binding motif derived from analyzing the 16 predicted NtrX binding sites listed in panel A.

Homologues of the NtrYX TCS have been identified across the bacterial domain, mostly in alpha- and betaproteobacteria (6). NtrX is typically annotated as a nitrogen regulation or assimilation protein; however, recent studies suggest that this is not a conserved function across bacterial species (6, 9). In this study, we show that NtrYX regulates the cell envelope in R. sphaeroides, while we did not find any evidence for regulation of nitrogen assimilation or metabolism. Genome-wide analysis for NtrX binding sites, similar to those performed here in R. sphaeroides, has not been reported in other bacteria, raising the question of whether there is a previously unrecognized role of this TCS in regulation of cell envelope more broadly across the phylogeny. To test this hypothesis, we analyzed potential genomic binding sites for NtrX in S. meliloti, where loss of NtrYX affects cell envelope properties (9), and in two other proteobacteria in which the NtrYX TCS is well-studied, B. abortus and Neisseria gonorrhoeae. R. sphaeroides NtrYX regulates the cell envelope by binding to sequences upstream of two operons in the conserved *dcw* gene cluster, *ddlA-ftsA* and *mraZ-mraY*, plus other genes (Table 1). To assess the possible presence of conserved NtrX binding sites, we compared the mapped NtrX binding site upstream of *ntrYX* in B. abortus (17), the sequences bound by this transcription factor in R. sphaeroides upstream of the *ddlA-ftsA* and *mraZ-mraY* operons (Table 1), and regions within 300 bp upstream of the *ddlA-ftsA* and *mraZ-mraY operons* in B. abortus, N. gonorrhoeae, and S. meliloti. This analysis revealed the existence of a common direct repeat sequence, GCAn9TGC, upstream of the first gene in each of these predicted operons (Fig. 6A). In addition, analysis of the genome-wide expression data available for a *ntrYX* mutant in S. meliloti revealed significant decreases in transcript levels (*P* < 0.05) for genes in the *ddlA-ftsA* and *mraZ-mraY* putative operons (9). Searching the other DNA sequences bound by R. sphaeroides NtrX revealed matches to this conserved binding sequence (Fig. 6A), allowing the development of a putative binding site for this NtrC family RR (Fig. 6B). Although additional research is needed to test the identity of this sequence as the NtrX binding site, this analysis suggests that the ability of NtrYX to regulate the cell envelope is conserved across bacteria.

### Indirect effects of the loss of NtrYX on the cell envelope.

We also observed a large increase in transcripts encoding the zinc-dependent metalloamidase LpxC in the Δ*ntrYX* strain compared to parent cells. Given the known regulatory role of LpxC in lipid A biosynthesis (42), is not surprising that we measured a reproducible increase in LPS levels in cells lacking NtrYX. We did not identify NtrX binding sites upstream of *lpxC* or other LPS biosynthesis genes, suggesting that LPS biosynthesis is indirectly regulated by this TCS. Other alterations in the cell envelope in cells lacking NtrYX are not clearly attributable to direct targets of NtrX or other transcriptome changes in cells lacking this TCS (increased phospholipid content, OM permeability, and periplasm thickness). Thus, with these pleotropic effects on the cell envelope, it is premature to propose a specific mechanistic model of how NtrX coordinates these direct and indirect effects on the cell envelope. Additional studies are needed to determine whether the changes in LPS levels, and other envelope alterations, are consequences of alterations in PG synthesis, the direct impact of NtrX on other putative transcription factors (MraZ), the action of additional target genes (RSP_1860, RSP_0332-35, RSP_2974-75) including an uncharacterized putative diguanylate cyclase/phosphodiesterase, or indirect effects of cell envelope perturbations in cells that lack NtrYX.

In conclusion, we have combined genetic, genomic, biochemical, and imaging studies to obtain important new insight into the transcriptional regulon for R. sphaeroides NtrX and shown that the NtrYX TCS has positive and negative effects on expression of genes that encode cell envelope functions. Thus, we conclude that in R. sphaeroides, NtrYX has a direct role in regulating assembly and function of the bacterial cell envelope. The R. sphaeroides HK NtrY amino acid sequence has a high degree of identity to B. abortus NtrY, which acts as a redox sensor by binding heme through its PAS domain and modulating the activity of the kinase based on the oxidation status of the heme iron. The amino acid identify between the PAS domain in the B. abortus and R. sphaeroides NtrY proteins (40% identical amino acids), along with the predicted PAC domain of R. sphaeroides (43), adjacent to the PAS domain, suggest that it is possible that R. sphaeroides NtrY also binds heme as a cofactor and is activated by a change in O_2_ tension. It is therefore plausible that the R. sphaeroides NtrYX TCS is involved in the remodeling of its cell envelope that is known to occur at low O_2_ tension. In the future, it will be important to elucidate the molecular impact of NtrYX on cell envelope biology, define how activity of this system is controlled, place this in the context of cell envelope process or regulators, and determine whether this TCS is associated with changes in extracytoplasmic functions in other species.

## MATERIALS AND METHODS

### Bacterial strains and growth conditions.

R. sphaeroides 2.4.1 strains used in this study Δ0382 (parent), ΔNtrYXΔ0382 (Δ*ntrYX*), and KL116, have been described previously (19). Cultures were grown in Sistrom’s minimal medium (SMM) (44), with 4 g/liter succinate, or where indicated 4.97 g/liter pyruvate (SMM-pyruvate). For aerobic cells, 10-ml cultures were grown in 125-ml flasks with shaking at ∼200 rpm at 30°C. For larger volumes, 500-ml cultures were grown in Roux bottles bubbled with 30% O_2_, 1% CO_2_, and 69% N_2_ (aerobic) or with 5% CO_2_ and 95% N_2_ (anaerobic). Anaerobic cultures were incubated in front of an incandescent light box with a light intensity of 10 W/m^2^ measured through a red glass filter. For N_2_-fixing conditions, a modified SMM was used without ammonium sulfate, glutamic acid, and aspartic acid.

### RNA extraction and transcriptome analysis.

R. sphaeroides cultures were grown aerobically in 500-ml cultures. At an optical density at 600 nm (OD_600_) of 0.3 to 0.4, 44 ml of harvested culture was combined with 6 ml of ice-cold stop solution (95% ethanol, 5% water-saturated phenol). These mixtures were centrifuged at 4°C for 10 min at 5,000 × *g*. Cell pellets were resuspended into 2 ml of lysis solution (2% sodium dodecyl sulfate [SDS], 16 mM EDTA in RNase-free water) and then incubated at 65°C for 5 min. Nucleic acid was extracted by adding equal volume of acid-phenol:chloroform (5:1; pH 4.5), mixing, incubating at 65°C for 5 min, mixing, and centrifuging at 16,000 × *g*. The aqueous phase was removed and extracted 2 more times, before adding an equal volume of chloroform, and centrifuging at 16,000 × *g* for 5 min. Then, 1/10 volume of 3 M sodium acetate and 1 ml isopropanol were added to the aqueous phase, followed by incubation at –20°C for 30 min, and then centrifugation at 16,000 × *g* for 30 min at 4°C to precipitate the nucleic acids. The pellet was washed with 75% ethanol and resuspended in RNase-free water. Samples were treated with RNase-free DNase and then purified with a RNeasy kit (Qiagen).

RNA-seq library preparation and sequencing were performed at the Joint Genome Institute. Libraries for sequencing were created using the Illumina TruSeq Stranded Total RNA kit (Illumina) following the standard protocol. RNA-seq libraries were sequenced on an Illumina NextSeq in 2x151 reads using the standard protocol. The paired-end FASTQ files were split into R1 and R2 files, and R1 files were retained for further analysis, as previous data contained only single-end reads. All FASTQ files were processed through the same pipeline. Reads were trimmed using Trimmomatic version 0.3 (45) with the default settings except for a HEADCROP of 5, LEADING of 3, TRAILING of 3, SLIDINGWINDOW of 3:30, and MINLEN of 36. After trimming, the reads were aligned to the R. sphaeroides 2.4.1 genome sequence (GenBank accession number GCA_000012905.2) using Bowtie2 version 2.2.2 (46) with default settings except that the number of mismatches was set at 1. Aligned reads were mapped to gene locations using HTSeq version 0.6.0 (47) using default settings except that the “reverse” strandedness argument was used. edgeR version 3.26.8 (48, 49) was used to identify significantly differentially expressed genes from pairwise analyses, using Benjamini and Hochberg (50) false discovery rate (FDR) of less than 0.05 as a significance threshold. Raw sequencing reads were normalized using the reads per kilobase per million mapped reads (RPKM). Pathway enrichment was performed using the SmartTable enrichment function at biocyc.org (51) using a *P* value of ≤0.05 as significant.

### Chromatin immunoprecipitation analysis.

R. sphaeroides KL116 cells, expressing isopropyl β-d-1-thiogalactopyranoside (IPTG)-inducible 3xMyc-tagged NtrX, were grown aerobically in 500-ml cultures. Cells were analyzed from two separate experiments using cultures grown with 3 or 50 μM IPTG at inoculation and harvested at mid-exponential phase. Chromatin immunoprecipitation (ChIP) was conducted as previously described (52), with polyclonal antibodies against the Myc epitope tag (ab9132; Abcam). Immunoprecipitated DNA was PCR amplified, gel purified (size selection, ∼200 bp) and sequenced at the University of Wisconsin-Madison Biotechnology Center sequencing facility, using a HiSeq 2500 sequencing system (Illumina, Inc.). The initial 50-bp sequence tags were mapped to the R. sphaeroides 2.4.1 genome (GenBank accession number GCA_000012905.2) using SOAP version 2.21 (53), allowing a maximum of two mismatches and no gaps. Peaks were identified using MOSAiCS (54) at a FDR of 0.05. The MOSAiCS analysis was conducted as a two-sample analysis, with ChIP-seq data from either input DNA or ChIP conducted with anti-Myc antibody in R. sphaeroides 2.4.1 without a Myc-tagged protein used as a control. Only peaks that were called significant using both controls (i.e., the intersect of the 2 analyses) were considered true peaks. The analysis was conducted using cells grown with 3 and 50 μM IPTG; Table 1 lists the peaks that were identified in both experiments, Data Set S2 in the supplemental material lists all the peaks identified in each replicate.

### Microscopy.

Bright-field microscopy images were acquired on a microscope EVOS FL auto with objective 100× oil immersion plan apochromat (numerical aperture, 1.40). Ten-milliliter aerobic cultures were grown until they reached an OD_600_ of 0.2 to 0.4 for exponential phase and an OD_600_ of >0.8 for stationary phase. Cells were placed on a coverglass under a 1.5% agarose pad. To facilitate cell segmentation, bright-field pictures were treated in FIJI with background subtraction (rolling ball radius = 30 pixels [pxs]), bandpass filter (large filter, 40 pxs; small filter, 2 pxs) and contrast enhancing (0.1%). Bright-field images were segmented by the plugin MicrobeJ (green contour in Fig. S1A and D) (22). The constriction feature of MicrobeJ (yellow bar in Fig. S1A) detects cells with a constriction at the mid-cell, corresponding to dividing cells. To improve accuracy of segmentation, membrane dye FM4-64 (Setareh Biotech) was used to verify the constriction rings and visualize the division septa, and DNA stain DAPI (Sigma-Aldrich) was used to assess for condensed DNA. Unpaired *t* tests were performed using software R.

TEM was performed as described previously (20), except that uranyl acetate was substituted with uranyl acetate alternative (Ted Pella). The periplasmic thickness was measured directly in Digital Micrograph in cell cross sections, as a perpendicular distance between the IM and OM.

### Analysis of PG precursors.

For hot water extracts, strains were grown aerobically for 3 days in 10-ml cultures in SMM-pyruvate and then diluted to an OD_600_ of 0.25 into 3 ml fresh medium in 25-ml Erlenmeyer flasks with 10-μCi/ml [^3^H]glucosamine. Cultures were grown for 5 h,  then washed twice with 3 ml medium, and added to 3 ml fresh medium. In order to determine the amount of incorporated label, the radioactivity of 30 μl of suspended culture was measured in counts per minute (cpm) by liquid scintillation counting. Cells were grown for an additional 45 min and then washed twice with 1 ml cold water, and the pellet was left on ice for 5 min. Pelleted cells were then suspended in 2 ml boiling water and then boiled for an additional 5 min. Samples were cooled to room temperature and centrifuged at 17,000 × *g* for 20 min. Soluble peptidoglycan precursors in the supernatant were then separated by size exclusion chromatography, and fractions were counted by liquid scintillation counting as described previously (55). Fraction cpm values were normalized to incorporated label of the parent strain.

### Cellular macromolecule measurements.

Ten-milliliter exponential-phase aerobic cultures were harvested by centrifuging at 10,000 × *g* for 15 min at 4°C, and the cell pellet was resuspended in an equal volume of medium. For protein, nucleic acid, and LPS measurements, the cells were lysed by sonicating on ice. Protein concentration was measured using the Pierce BCA protein assay kit (ThermoFisher Scientific), and nucleic acids were measured using a Qubit fluorimeter using the 1× dsDNA HS assay kit and the RNA BR assay kit (Invitrogen). LPS was measured using the Pierce LAL chromogenic endotoxin quantification kit (ThermoFisher Scientific) with samples serially diluted in endotoxin-free water. All kits were used according to the manufacturers’ protocols. Lipid extraction with chloroform-methanol, esterification, gas chromatography-mass spectrometry (GC-MS) analysis, and quantification (20) were performed using 2.5-ml samples. The measured fatty acid levels were converted using the average phospholipid molecular weight of 718.1 g/mol, which takes into account the relative abundance of the fatty acid species that we measure, and the reported composition of the three most abundant phospholipids in R. sphaeroides (56, 57).

Absorbance scans to detect pigment-protein complexes were performed on 200 μl of aerobic culture in Nunc 96-well black optical-bottom plates (ThermoFisher Scientific), using an Infinite M1000 plate reader (Tecan). Strains and growth conditions that contain pigment-protein complexes (20) were used as positive controls.

### Cell permeability assays.

OM permeability was measured on exponential cells (OD_600_ of 0.3 to 0.5) grown in 10-ml aerobic cultures, washed, and resuspended with 5 mM HEPES, and normalized to an OD_600_ of 0.5. NPN uptake assays were performed as described previously (30), using a final concentration of 5 μM NPN. Fluorescence measurements (excitation, 340 nm; emission, 400 nm) were taken on an Infinite M1000 plate reader (Tecan). Background fluorescence of NPN only (no cells) was subtracted from all fluorescence measurements and then scaled relative to untreated parent cells.

PI staining was measured on cell samples prepared as for NPN assays, except that phosphate-buffered saline (PBS) was used to wash and resuspend the cells. PI (1.0 mg/ml in water) (ThermoFisher Scientific) was diluted to 10 μg/ml in PBS. To perform the assays, 10 μl Triton X-100 (0.2% or 2%), 20 μl PI, and 170 μl cell solution were combined in Nunc 96-well black optical-bottom plates (ThermoFisher Scientific). Fluorescence (excitation 535 nm, emission 625 nm) was read immediately, and calculations were performed as for NPN assays.

### NtrX binding site analysis.

To identify potential NtrX binding sequences, we compared the sequence identified from foot-printing full-length wild-type NtrX on the *ntrY* promoter in B. abortus (17) to known binding sequences for NtrC family transcription factors (58–62), since NtrX is a member of the NtrC subfamily of RRs (17). The common sequence pattern that emerged from this comparison was GCAnnnnnnnnnTGC, so this was used to search sequences 300 bp upstream of the ATG start codon for the first gene in each predicted operon for potential NtrX binding sequences. We first searched the seven sites known to be bound by R. sphaeroides NtrX (Table 1) and the B. abortus *ntrY* promoter (17). We also examined the DNA upstream of *ntrY* in N. gonorrhoeae and S. meliloti and that upstream of the predicted *dcw* gene cluster operons *ddlA-ftsA* and *mraZ-mraY* in B. abortus, N. gonorrhoeae, and S. meliloti for NtrX binding sites. The single best potential NtrX binding sequence in each of these regions was identified, and a logo was derived from these 16 potential binding sites using WebLogo (https://weblogo.berkeley.edu).

### Data availability.

The RNA-seq and ChIP-seq data are available in the National Center for Biotechnology Information Gene Expression Omnibus under accession number GSE145442.

## References

[1] Silhavy TJ, Kahne D, Walker S 2010 The bacterial cell envelope. Cold Spring Harb Perspect Biol 2:a000414. doi:10.1101/cshperspect.a000414. 20452953 PMC2857177

[2] Wyckoff TJ, Taylor JA, Salama NR 2012 Beyond growth: novel functions for bacterial cell wall hydrolases. Trends Microbiol 20:540–547. doi:10.1016/j.tim.2012.08.003. 22944244 PMC3479350

[3] Rowley G, Spector M, Kormanec J, Roberts M 2006 Pushing the envelope: extracytoplasmic stress responses in bacterial pathogens. Nat Rev Microbiol 4:383–394. doi:10.1038/nrmicro1394. 16715050

[4] Zschiedrich CP, Keidel V, Szurmant H 2016 Molecular mechanisms of two-component signal transduction. J Mol Biol 428:3752–3775. doi:10.1016/j.jmb.2016.08.003. 27519796 PMC5023499

[5] Cardona ST, Choy M, Hogan AM 2018 Essential two-component systems regulating cell envelope functions: opportunities for novel antibiotic therapies. J Membr Biol 251:75–89. doi:10.1007/s00232-017-9995-5. 29098331

[6] Atack JM, Srikhanta YN, Djoko KY, Welch JP, Hasri NH, Steichen CT, Vanden Hoven RN, Grimmond SM, Othman DS, Kappler U, Apicella MA, Jennings MP, Edwards JL, McEwan AG 2013 Characterization of an *ntrX* mutant of *Neisseria gonorrhoeae* reveals a response regulator that controls expression of respiratory enzymes in oxidase-positive proteobacteria. J Bacteriol 195:2632–2641. doi:10.1128/JB.02062-12. 23564168 PMC3676050

[7] Carrica MDC, Fernandez I, Marti MA, Paris G, Goldbaum FA 2012 The NtrY/X two-component system of *Brucella* spp. acts as a redox sensor and regulates the expression of nitrogen respiration enzymes. Mol Microbiol 85:39–50. doi:10.1111/j.1365-2958.2012.08095.x. 22582926

[8] Carrica MDC, Fernandez I, Sieira R, Paris G, Goldbaum FA 2013 The two-component systems PrrBA and NtrYX co-ordinately regulate the adaptation of *Brucella abortus* to an oxygen-limited environment. Mol Microbiol 88:222–233. doi:10.1111/mmi.12181. 23527685

[9] Calatrava-Morales N, Nogales J, Ameztoy K, van Steenbergen B, Soto MJ 2017 The NtrY/NtrX system of *Sinorhizobium meliloti* GR4 regulates motility, EPS I production, and nitrogen metabolism but is dispensable for symbiotic nitrogen fixation. Mol Plant Microbe Interact 30:566–577. doi:10.1094/MPMI-01-17-0021-R. 28398840

[10] Pawlowski K, Klosse U, de Bruijn FJ 1991 Characterization of a novel *Azorhizobium caulinodans* ORS571 two-component regulatory system, NtrY/NtrX, involved in nitrogen fixation and metabolism. Mol Gen Genet 231:124–138. doi:10.1007/bf00293830. 1661370

[11] López MF, Hegel VA, Torres MJ, García AH, Delgado MJ, López-García SL 2019 The *Bradyrhizobium diazoefficiens* two-component system NtrYX has a key role in symbiotic nitrogen fixation of soybean plants and *cbb*_3_ oxidase expression in bacteroids. Plant Soil 440:167–183. doi:10.1007/s11104-019-04067-0.

[12] Nogales J, Campos R, BenAbdelkhalek H, Olivares J, Lluch C, Sanjuan J 2002 *Rhizobium tropici* genes involved in free-living salt tolerance are required for the establishment of efficient nitrogen-fixing symbiosis with *Phaseolus vulgaris*. Mol Plant Microbe Interact 15:225–232. doi:10.1094/MPMI.2002.15.3.225. 11952125

[13] Cheng Z, Lin M, Rikihisa Y 2014 *Ehrlichia chaffeensis* proliferation begins with NtrY/NtrX and PutA/GlnA upregulation and CtrA degradation induced by proline and glutamine uptake. mBio 5:e02141-14. doi:10.1128/mBio.02141-14. 25425236 PMC4251998

[14] Gregor J, Zeller T, Balzer A, Haberzettl K, Klug G 2007 Bacterial regulatory networks include direct contact of response regulator proteins: interaction of RegA and NtrX in *Rhodobacter capsulatus*. J Mol Microbiol Biotechnol 13:126–139. doi:10.1159/000103604. 17693720

[15] Wang D, Xue H, Wang Y, Yin R, Xie F, Luo L 2013 The *Sinorhizobium meliloti ntrX* gene is involved in succinoglycan production, motility, and symbiotic nodulation on alfalfa. Appl Environ Microbiol 79:7150–7159. doi:10.1128/AEM.02225-13. 24038694 PMC3837732

[16] Bonato P, Alves LR, Osaki JH, Rigo LU, Pedrosa FO, Souza EM, Zhang N, Schumacher J, Buck M, Wassem R, Chubatsu LS 2016 The NtrY-NtrX two-component system is involved in controlling nitrate assimilation in *Herbaspirillum seropedicae* strain SmR1. FEBS J 283:3919–3930. doi:10.1111/febs.13897. 27634462

[17] Fernández I, Cornaciu I, Carrica MDC, Uchikawa E, Hoffmann G, Sieira R, Márquez JA, Goldbaum FA 2017 Three-dimensional structure of full-length NtrX, an unusual member of the NtrC family of response regulators. J Mol Biol 429:1192–1212. doi:10.1016/j.jmb.2016.12.022. 28088479

[18] Tavano CL, Donohue TJ 2006 Development of the bacterial photosynthetic apparatus. Curr Opin Microbiol 9:625–631. doi:10.1016/j.mib.2006.10.005. 17055774 PMC2765710

[19] Lemmer KC, Zhang W, Langer SJ, Dohnalkova AC, Hu D, Lemke RA, Piotrowski JS, Orr G, Noguera DR, Donohue TJ 2017 Mutations that alter the bacterial cell envelope increase lipid production. mBio 8:e00513-17. doi:10.1128/mBio.00513-17. 28536286 PMC5442454

[20] Lemmer KC, Dohnalkova AC, Noguera DR, Donohue TJ 2015 Oxygen-dependent regulation of bacterial lipid production. J Bacteriol 197:1649–1658. doi:10.1128/JB.02510-14. 25733615 PMC4403652

[21] Harry E, Monahan L, Thompson L 2006 Bacterial cell division: the mechanism and its precison. Int Rev Cytol 253:27–94. doi:10.1016/S0074-7696(06)53002-5. 17098054

[22] Ducret A, Quardokus EM, Brun YV 2016 MicrobeJ, a tool for high throughput bacterial cell detection and quantitative analysis. Nat Microbiol 1:16077. doi:10.1038/nmicrobiol.2016.77. 27572972 PMC5010025

[23] Typas A, Banzhaf M, Gross CA, Vollmer W 2011 From the regulation of peptidoglycan synthesis to bacterial growth and morphology. Nat Rev Microbiol 10:123–136. doi:10.1038/nrmicro2677. 22203377 PMC5433867

[24] van Teeseling MCF, de Pedro MA, Cava F 2017 Determinants of bacterial morphology: from fundamentals to possibilities for antimicrobial targeting. Front Microbiol 8:1264. doi:10.3389/fmicb.2017.01264. 28740487 PMC5502672

[25] Pisabarro AG, de Pedro MA, Vazquez D 1985 Structural modifications in the peptidoglycan of *Escherichia coli* associated with changes in the state of growth of the culture. J Bacteriol 161:238–242. doi:10.1128/JB.161.1.238-242.1985. 3881387 PMC214862

[26] Barreteau H, Kovač A, Boniface A, Sova M, Gobec S, Blanot D 2008 Cytoplasmic steps of peptidoglycan biosynthesis. FEMS Microbiol Rev 32:168–207. doi:10.1111/j.1574-6976.2008.00104.x. 18266853

[27] Schaub RE, Chan YA, Lee M, Hesek D, Mobashery S, Dillard JP 2016 Lytic transglycosylases LtgA and LtgD perform distinct roles in remodeling, recycling and releasing peptidoglycan in *Neisseria gonorrhoeae*. Mol Microbiol 102:865–881. doi:10.1111/mmi.13496. 27608412 PMC5463997

[28] Cheng Q, Li H, Merdek K, Park JT 2000 Molecular characterization of the beta-N-acetylglucosaminidase of *Escherichia coli* and its role in cell wall recycling. J Bacteriol 182:4836–4840. doi:10.1128/jb.182.17.4836-4840.2000. 10940025 PMC111361

[29] Nikaido H 2003 Molecular basis of bacterial outer membrane permeability revisited. Microbiol Mol Biol Rev 67:593–656. doi:10.1128/mmbr.67.4.593-656.2003. 14665678 PMC309051

[30] Helander IM, Mattila-Sandholm T 2000 Fluorometric assessment of Gram-negative bacterial permeabilization. J Appl Microbiol 88:213–219. doi:10.1046/j.1365-2672.2000.00971.x. 10735988

[31] Vitorino JC, Steffens MB, Machado HB, Yates MG, Souza EM, Pedrosa FO 2001 Potential roles for the *glnB* and *ntrYX* genes in *Azospirillum brasilense*. FEMS Microbiol Lett 201:199–204. doi:10.1111/j.1574-6968.2001.tb10757.x. 11470362

[32] Bush M, Dixon R 2012 The role of bacterial enhancer binding proteins as specialized activators of σ^54^-dependent transcription. Microbiol Mol Biol Rev 76:497–529. doi:10.1128/MMBR.00006-12. 22933558 PMC3429621

[33] Lee TK, Huang KC 2013 The role of hydrolases in bacterial cell-wall growth. Curr Opin Microbiol 16:760–766. doi:10.1016/j.mib.2013.08.005. 24035761 PMC3986426

[34] Sycuro LK, Pincus Z, Gutierrez KD, Biboy J, Stern CA, Vollmer W, Salama NR 2010 Peptidoglycan crosslinking relaxation promotes *Helicobacter pylori*’s helical shape and stomach colonization. Cell 141:822–833. doi:10.1016/j.cell.2010.03.046. 20510929 PMC2920535

[35] Hay NA, Tipper DJ, Gygi D, Hughes C 1999 A novel membrane protein influencing cell shape and multicellular swarming of *Proteus mirabilis*. J Bacteriol 181:2008–2016. doi:10.1128/JB.181.7.2008-2016.1999. 10094676 PMC93611

[36] Ayala JA, Garrido T, De Pedro MA, Vicente M 1994 Chapter 5. Molecular biology of bacterial septation, p 73–101. *In* Ghuysen J-M, Hakenbeck R (ed), New comprehensive biochemistry, vol 27 Bacterial cell wall. Elsevier Science, Amsterdam, The Netherlands.

[37] Vicente M, Gomez MJ, Ayala JA 1998 Regulation of transcription of cell division genes in the *Escherichia coli dcw* cluster. Cell Mol Life Sci 54:317–324. doi:10.1007/s000180050158. 9614967 PMC11147420

[38] Egan AJ, Vollmer W 2013 The physiology of bacterial cell division. Ann N Y Acad Sci 1277:8–28. doi:10.1111/j.1749-6632.2012.06818.x. 23215820

[39] Eraso JM, Markillie LM, Mitchell HD, Taylor RC, Orr G, Margolin W 2014 The highly conserved MraZ protein is a transcriptional regulator in *Escherichia coli*. J Bacteriol 196:2053–2066. doi:10.1128/JB.01370-13. 24659771 PMC4010979

[40] Mura A, Fadda D, Perez AJ, Danforth ML, Musu D, Rico AI, Krupka M, Denapaite D, Tsui H-CT, Winkler ME, Branny P, Vicente M, Margolin W, Massidda O 2017 Roles of the essential protein FtsA in cell growth and division in Streptococcus pneumoniae. J Bacteriol 199:e00608-16. doi:10.1128/JB.00608-16. 27872183 PMC5237122

[41] Jain P, Malakar B, Khan MZ, Lochab S, Singh A, Nandicoori VK 2018 Delineating FtsQ mediated regulation of cell division in *Mycobacterium tuberculosis*. J Biol Chem 293:12331–12349. doi:10.1074/jbc.RA118.003628. 29903917 PMC6093227

[42] Pei Z, Adam WB 2008 Mechanism and inhibition of LpxC: an essential zinc-dependent deacetylase of bacterial lipid A synthesis. Curr Pharm Biotechnol 9:9–15. doi:10.2174/138920108783497668. 18289052 PMC3022321

[43] The UniProt Consortium. 2019 UniProt: a worldwide hub of protein knowledge. Nucleic Acids Res 47:D506–D515. doi:10.1093/nar/gky1049. 30395287 PMC6323992

[44] Sistrom WR 1960 A requirement for sodium in the growth of *Rhodopseudomonas spheroides*. J Gen Microbiol 22:778–785. doi:10.1099/00221287-22-3-778. 14447230

[45] Bolger AM, Lohse M, Usadel B 2014 Trimmomatic: a flexible trimmer for Illumina sequence data. Bioinformatics 30:2114–2120. doi:10.1093/bioinformatics/btu170. 24695404 PMC4103590

[46] Langmead B, Salzberg SL 2012 Fast gapped-read alignment with Bowtie 2. Nat Methods 9:357–359. doi:10.1038/nmeth.1923. 22388286 PMC3322381

[47] Anders S, Pyl PT, Huber W 2015 HTSeq–a Python framework to work with high-throughput sequencing data. Bioinformatics 31:166–169. doi:10.1093/bioinformatics/btu638. 25260700 PMC4287950

[48] Robinson MD, McCarthy DJ, Smyth GK 2010 edgeR: a Bioconductor package for differential expression analysis of digital gene expression data. Bioinformatics 26:139–140. doi:10.1093/bioinformatics/btp616. 19910308 PMC2796818

[49] McCarthy DJ, Chen Y, Smyth GK 2012 Differential expression analysis of multifactor RNA-Seq experiments with respect to biological variation. Nucleic Acids Res 40:4288–4297. doi:10.1093/nar/gks042. 22287627 PMC3378882

[50] Benjamini Y, Hochberg Y 1995 Controlling the false discovery rate: a practical and powerful approach to multiple testing. J R Stat Soc Series B Stat Methodol 57:289–300. doi:10.1111/j.2517-6161.1995.tb02031.x.

[51] Karp PD, Billington R, Caspi R, Fulcher CA, Latendresse M, Kothari A, Keseler IM, Krummenacker M, Midford PE, Ong Q, Ong WK, Paley SM, Subhraveti P 2019 The BioCyc collection of microbial genomes and metabolic pathways. Brief Bioinform 20:1085–1093. doi:10.1093/bib/bbx085. 29447345 PMC6781571

[52] Dufour YS, Landick R, Donohue TJ 2008 Organization and evolution of the biological response to singlet oxygen stress. J Mol Biol 383:713–730. doi:10.1016/j.jmb.2008.08.017. 18723027 PMC2579311

[53] Li R, Yu C, Li Y, Lam TW, Yiu SM, Kristiansen K, Wang J 2009 SOAP2: an improved ultrafast tool for short read alignment. Bioinformatics 25:1966–1967. doi:10.1093/bioinformatics/btp336. 19497933

[54] Kuan PF, Chung D, Pan G, Thomson JA, Stewart R, Keles S 2011 A statistical framework for the analysis of ChIP-Seq data. J Am Stat Assoc 106:891–903. doi:10.1198/jasa.2011.ap09706. 26478641 PMC4608541

[55] Cloud KA, Dillard JP 2002 A lytic transglycosylase of *Neisseria gonorrhoeae* is involved in peptidoglycan-derived cytotoxin production. Infect Immun 70:2752–2757. doi:10.1128/iai.70.6.2752-2757.2002. 12010959 PMC127960

[56] Russell NJ, Harwood JL 1979 Changes in the acyl lipid composition of photosynthetic bacteria grown under photosynthetic and non-photosynthetic conditions. Biochem J 181:339–345. doi:10.1042/bj1810339. 115463 PMC1161166

[57] Marinetti GV, Cattieu K 1981 Lipid analysis of cells and chromatophores of *Rhodopseudomonas sphaeroides*. Chem Phys Lipids 28:241–251. doi:10.1016/0009-3084(81)90011-6.

[58] Contreras A, Drummond M 1988 The effect on the function of the transcriptional activator NtrC from *Klebsiella pneumoniae* of mutations in the DNA-recognition helix. Nucleic Acids Res 16:4025–4039. doi:10.1093/nar/16.9.4025. 3287338 PMC336572

[59] Ferro-Luzzi Ames G, Nikaido K 1985 Nitrogen regulation in *Salmonella typhimurium*. Identification of an ntrC protein-binding site and definition of a consensus binding sequence. EMBO J 4:539–547. doi:10.1002/j.1460-2075.1985.tb03662.x. 2862031 PMC554219

[60] Robison K, McGuire AM, Church GM 1998 A comprehensive library of DNA-binding site matrices for 55 proteins applied to the complete *Escherichia coli* K-12 genome. J Mol Biol 284:241–254. doi:10.1006/jmbi.1998.2160. 9813115

[61] Spinosa M, Riccio A, Mandrich L, Manco G, Lamberti A, Iaccarino M, Merrick M, Patriarca EJ 2000 Inhibition of glutamine synthetase II expression by the product of the *gstI* gene. Mol Microbiol 37:443–452. doi:10.1046/j.1365-2958.2000.02018.x. 10931338

[62] De Zamaroczy M, Delorme F, Elmerich C 1990 Characterization of three different nitrogen-regulated promoter regions for the expression of *glnB* and *glnA* in *Azospirillum brasilense*. Mol Gen Genet 224:421–430. doi:10.1007/bf00262437. 1702507

