# Supplementary material for: The NtrYX Two-Component System Regulates the Bacterial Cell Envelope
Source: mBio. 2020 May 19;11(3):e00957-20. doi: 10.1128/mBio.00957-20 (PMC7240162; doi:10.1128/mBio.00957-20)
Supplement: TABLE S1 [file mBio.00957-20-st001.pdf]

Table S1 Growth rates of parent and Δ*ntrYX* cultures

Growth condition	Doubling time (hours)		Relative change	n	p value
	Parent	Δ*ntrYX*			
Aerobic, NH_4	2.9 ± 0.3	4.0 ± 0.4	+ 41%	6	0.0002
Anaerobic + light, NH_4	3.6 ± 0.5	5.9 ± 1.2	+ 65%	7	0.0004
Anaerobic + light, N_2	7.1 ± 0.6	8.9 ± 1.3	+ 25%	6	0.0119
