# Supplementary figures and images for: The NtrYX Two-Component System Regulates the Bacterial Cell Envelope
Source: mBio. 2020 May 19;11(3):e00957-20. doi: 10.1128/mBio.00957-20 (PMC7240162; doi:10.1128/mBio.00957-20)

**A**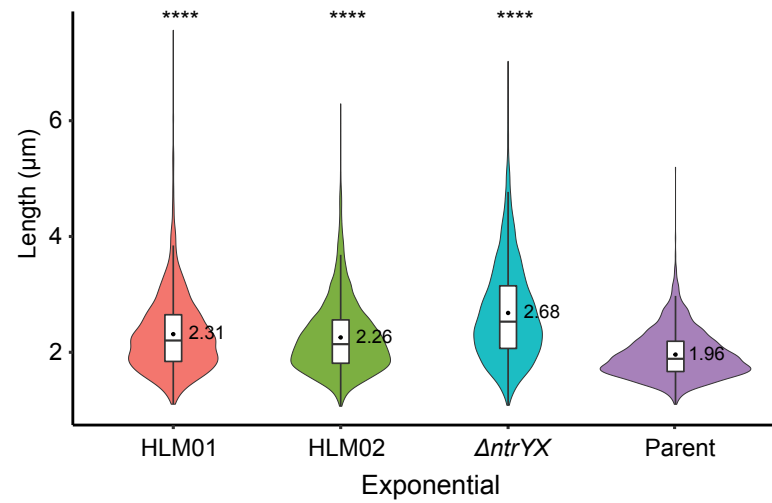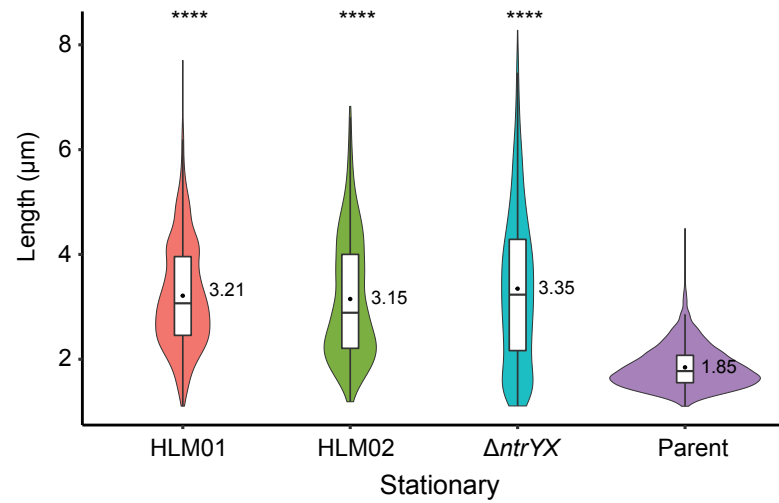**B**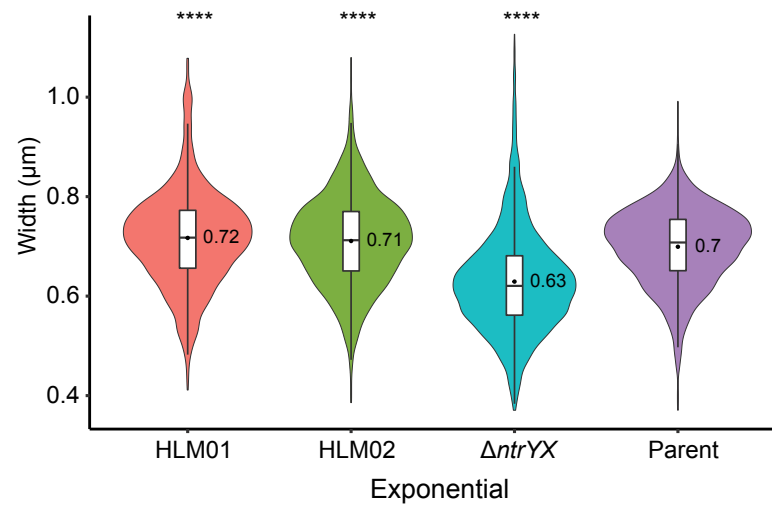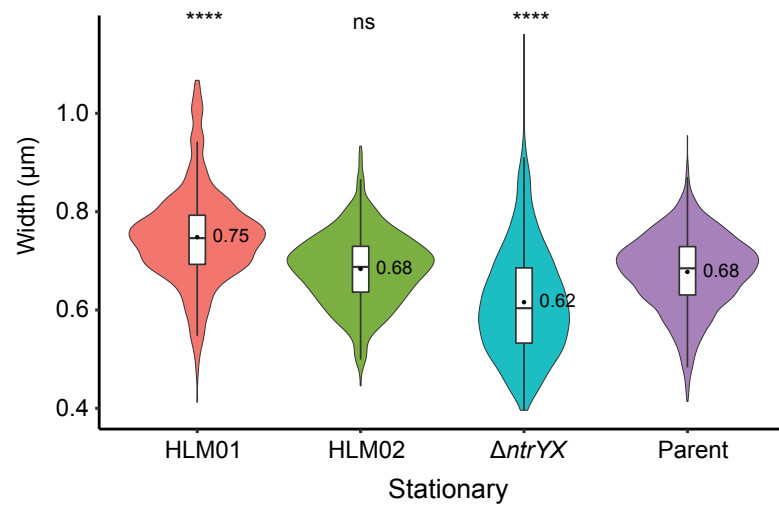**C**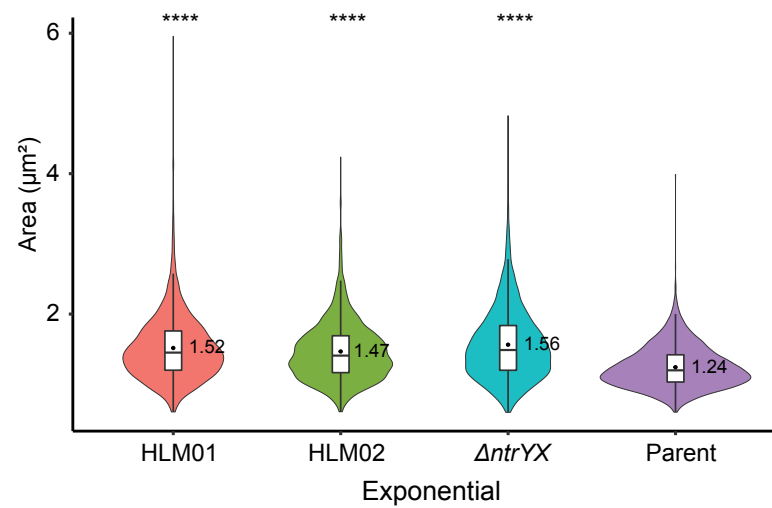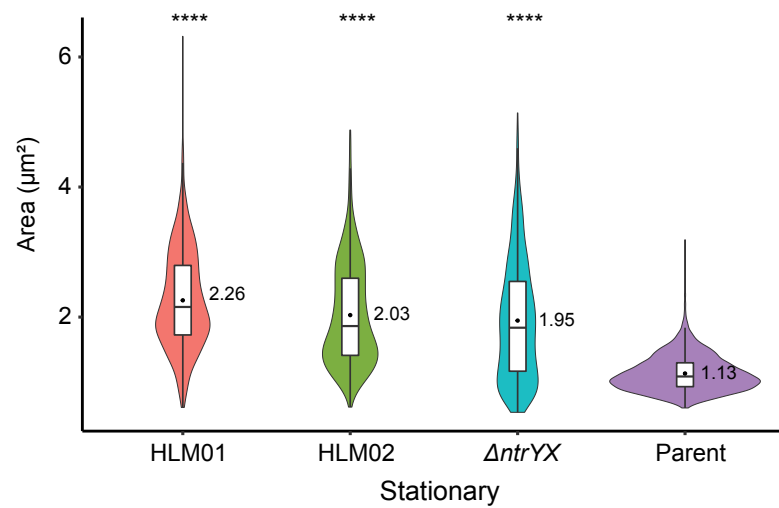

Supplement: FIG S2 [file mBio.00957-20-sf002.pdf]

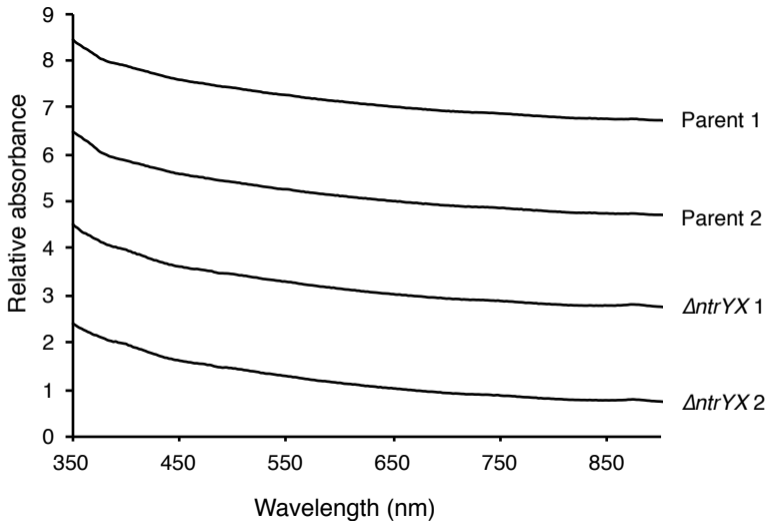

Supplement: FIG S4 [file mBio.00957-20-sf004.pdf]
