# Supplementary material for: The NtrYX Two-Component System Regulates the Bacterial Cell Envelope
Source: mBio. 2020 May 19;11(3):e00957-20. doi: 10.1128/mBio.00957-20 (PMC7240162; doi:10.1128/mBio.00957-20)
Supplement: TABLE S2 [file mBio.00957-20-st002.pdf]

Table S2 Cell biomass composition in μg/ml of aerobically grown parent and *ΔntrYX* cultures, both normalized to an absorbance of 1 at 600 nm

Strain	Phospholipid	LPS	Protein	DNA	RNA
Parent	25.7 ± 3.0	3.9 ± 0.9	235 ± 21	18.1 ± 5.4	30.2 ± 5.3
ΔntrYX	53.0 ± 7.9[a]	8.0 ± 2.6[b]	229 ± 18	17.7 ± 1.7	31.3 ± 2.4

[a] $p < 0.0001$ compared to parent cells
[b] $p < 0.001$ compared to parent cells
