# Supplementary material for: The NtrYX Two-Component System Regulates the Bacterial Cell Envelope
Source: mBio. 2020 May 19;11(3):e00957-20. doi: 10.1128/mBio.00957-20 (PMC7240162; doi:10.1128/mBio.00957-20)
Supplement: TABLE S3 [file mBio.00957-20-st003.pdf]

Table S3 Growth of parent and *ΔntrYX* strains with different nitrogen sources

		N source			
Strain	Growth condition	NH_4	Urea	Glutamate	N_2
Parent	Aerobic[a]	++	+	++	nd
	Anaerobic + light[b]	++	++	++	++
ΔntrYX	Aerobic[a]	++	+	++	nd
	Anaerobic + light[b]	++	++	++	++

++, strain grew well; +, strain grew poorly; nd, not done.

[a] Aerobic cultures were grown in 200 µl of SMM in 96-well plates at 30 °C with shaking in an Infinite M1000 plate reader (Tecan).

[b] Anaerobic cultures were grown in sealed ~18 ml screw caps tubes incubated at room temperature in front of an incandescent light with a light intensity of 10 W/m^2 measured through a red glass filter.
